# Simulating the Performance of a Formamidinium Based Mixed Cation Lead Halide Perovskite Solar Cell

**DOI:** 10.3390/ma14216341

**Published:** 2021-10-23

**Authors:** Denis Stanić, Vedran Kojić, Tihana Čižmar, Krunoslav Juraić, Lara Bagladi, Jimmy Mangalam, Thomas Rath, Andreja Gajović

**Affiliations:** 1 Department of Physics, Josip Juraj Strossmayer University of Osijek, Trg Ljudevita Gaja 6, 31000 Osijek, Croatia; dstanic@fizika.unios.hr; 2 Ruđer Bošković Institute, Bijenička Cesta 54, 10000 Zagreb, Croatia; vkojic@irb.hr (V.K.); tcizmar@irb.hr (T.Č.); kjuraic@irb.hr (K.J.); 3 Faculty of Chemical Engineering and Technology, University of Zagreb, Marulićev trg 19, 10000 Zagreb, Croatia; larabagladi@gmail.com; 4 Institute for Chemistry and Technology of Materials (ICTM), NAWI Graz, Graz University of Technology, Stremayrgasse 9/V, A-8010 Graz, Austria; jimmy.mangalam@ddn.upes.ac.in (J.M.); thomas.rath@tugraz.at (T.R.); 5 Department of Chemistry, School of Engineering, University of Petroleum and Energy Studies, Bidholi Via Prremnagar, Dehradun 248007, Uttarakhand, India

**Keywords:** perovskite solar cells, device simulation, SCAPS-1D, power conversion efficiency

## Abstract

With the aim of decreasing the number of experiments to obtain a perovskite solar cell (PSC) with maximum theoretical efficiency, in this paper, PSC performance was studied using the program solar cell capacitance simulator (SCAPS-1D). The PSC with the architecture ITO/TiO_2_/perovskite/spiro-MeOTAD/Au was investigated, while the selected perovskite was mixed cation Rb_0.05_Cs_0.1_FA_0.85_PbI_3_. The analysis was based on an experimentally prepared solar cell with a power conversion efficiency of ~7%. The PSC performance, verified by short-circuit current density (*J*_sc_), open-circuit voltage (*V*_oc_), fill factor (*FF*) and power conversion efficiency (*PCE*), was studied by optimization of the simulation parameters responsible for improvement of the cell operation. The optimized parameters were absorber layer thickness, doping, defect concentration and the influence of the resistivity (the net effect of ohmic loss, *R_s_* and the leakage current loss represented by the resistivity, *R_shunt_*). The results of SCAPS-1D simulations estimated the theoretical power conversion efficiency of 15% for our material. We have showed that the main contribution to improvement of solar cell efficiency comes with lowering ohmic resistivity of the cell as well as doping and defect concentration, because their concentration is proportional to recombination rate.

## 1. Introduction

Since their discovery in 2009 [1,2,3], perovskite solar cells (PSCs) have drawn great attention. Besides their low-cost solution processing, PSCs also possess favourable optoelectronic properties including a high absorption coefficient in the visible part of solar spectrum [4], low recombination rate [5], high mobility of charge carriers [6] and a tuneable bandgap [7]. In the past decade, their power conversion efficiency (*PCE*) has been significantly enhanced from 3.8% in 2009 [1] to more than 25% in single-junction architectures and more than 29% in silicon-based tandem cells [8,9,10]. The enhancement of the *PCE*, one of the key factors of solar cells, defines PSCs as the promising candidates for commercialization in the solar cell industry. Furthermore, many efforts have been made to develop advanced structures of perovskites [11] for applications in photolysis, photodetectors [12] and various type of solar cells such as flexible cells [13], carbon-electrode-based cells [14], semi-transparent cells [15], tandem cells [16], integrated cells [17], switchable cells [18], and single crystal cells [19]. Perovskite solar cells are photovoltaics with solution processed active layer, one of several recently studied technology. The other broadly investigated photovoltaics with solution processed active layer, include photovoltaics based on the organic semiconductors as active layers [20,21,22]. All of these PSC devices are supposed to have high power efficiency, high stability, and large-scale applicability. However, there are still several issues in practical operations of PSCs, such as the long-term instability due to the perovskite degradation (moisture, UV light, overheating) [11,23,24], the toxicity of the most commonly used compounds [25], and the current-voltage hysteresis in devices [26]. In order to solve these obstacles, many methods have been used, such as doping ions in perovskite materials, charge transporting layer modification, modification of microstructure, and utilizing advanced fabrication techniques [11].

The mostly used perovskite materials are methylammonium lead triiodide (MAPbI_3_) and formamidinium lead triiodide (FAPbI_3_), and their use as the absorber layer in PSCs has been extensively studied [27,28]. The results have shown that FAPbI_3_ is thermally more stable, which makes it the most promising perovskite material for single-junction PSCs. Recently, rubidium and cesium cation (Rb^+^ and Cs^+^) incorporation emerged as a strategy to enhance PSCs efficiency [29].

In conjunction with the perovskite materials as the photoactive absorber layer, other thin film materials are used to extract photogenerated electrons and holes, reducing charge recombination inside the absorber layer and improving the overall efficiency of the solar cell. Indeed, such layers have their energy band levels aligned in a way which creates a potential difference that drives the charge separation. For the charge separation of electrons, the material has to have lower conduction band (CB) maximum and valence band (VB) minimum energy levels than those of the absorber layer in order to promote electron extraction and block hole extraction from the active layer. These materials are called electron transport layer (ETLs) and most commonly used are TiO_2_, ZnO and SnO_2_ [30]. Analogous to ETLs, materials which have higher CB maximum and VB minimum energy levels in comparison to those levels of the absorber layer, promote hole extraction and block electron extraction. These layers are called hole transport layers (HTLs) and the most popular are spiro-OMeTAD, P3HT, PTTA and PEDOT:PPS organic semiconductors [31].

Since the main aim of numerous experimental studies is increase of the device *PCE*, it is worth theoretically studying the influence of the specific parameters on the performance of the PSC by applying an appropriate model. In this work, we have employed one dimensional theoretical model of solar cell integrated in the solar cell capacitance simulator (SCAPS-1D) [32] in order to elucidate which experimental procedure could improve the PSC parameters. The parameters that we varied by SCAPS-1D simulation and that can be influenced experimentally are: (1) acceptor doping concentration (*N*_A_) of the perovskite absorber layer, (2) defect density in the perovskite absorber layer, (3) the thickness of the perovskite absorption layer, (4) resistivity *R_s_* (the net effect of ohmic loss) and *R_shunt_* (the leakage current loss).

The first mentioned parameter, which can be influenced experimentally, is doping concentration *N*_A_. There are several studies which attempted to investigate how to influence the charge carrier concentration during the synthesis of the perovskite thin films. Shi and co-workers [33] investigated the influence of precursor concertation and solvent choice in a two-step deposition method of the absorber layer. They reported that the carrier density and the depletion field decrease with the increase of the concentration of CH_3_NH_3_PbI_3_ immersion solution (CH_3_NH_3_I dissolved in isopropanol), and also showed that the inclusion of a polar solvent improved the charge collection by influencing the reaction kinetics for the formation of the perovskite films. Bi et al. [34] investigated the role of the length of thermal annealing and show that the extended annealing time improved the crystallinity and grain size of the perovskite crystals, but also led to the reduction of hole concentration. While improved crystallinity led to better *FF* and *J*_sc_ values, ultraviolet photoelectron spectroscopy studies confirmed the reduction of the work function with increased the annealing time. Several authors also reported the effects of adding the cations or the anions in the perovskite active layer and its impact on carrier concentrations. Liu and co-workers [35] introduced rubidium cations into the perovskite film. While the crystal structure remained the same, the quality of the film worsened, reducing the efficiency of the device. However, rubidium cations made the majority carrier type of the perovskite layer into p-type, and previously formed pn junction between the active layer and the spiro-MeOTAD layer was replaced with a new pn junction between the active layer and TiO_2_. The influence of the anion composition was investigated by Kiermasch et al. [36], who doped the MAPbI_3_ perovskite with bromine anions and concluded that bromine’s addition did not influence the carrier concentration, but instead increased carrier lifetime. They hypothesize that the origin of the enhancement lies in the increase in shallow traps close to the conduction or valence band. These states partially trap charge carriers which can then be released back into the band before they recombine, leading to the increase of the *V*_oc_ while *J*_sc_ and *FF* both decreased.

Second parameter, density of defects, are mainly influenced by the nature of the crystallization of the perovskite thin film. The origin of the defects resides on the grain boundaries of the polycrystalline films as well as dangling bonds, uncoordinated atoms and surface dislocation on the film surface. Influencing the crystallization process, the amount of defect densities that occur in perovskite thin films can be altered. Tan et al. [37] employed an additional post annealing treatment of the methylammonium chloride perovskite films. Those films changed their microstructural orientation from the randomly oriented to the preferred (110) crystal plane growth, made possible by exploiting the chlorine intermediate phase analogous to the mineral bridge phenomenon in the oriented attachment mechanism. *FF*, *J*_sc_ and *V*_oc_ were improved as a result of lowered trap state densities with regards to oriented crystal growth [38]; indeed, another way to impact the crystallization mechanism is through additive engineering. Yang and associates [39] used the ammonium benzenesulfonate as an active layer additive; its zwitterionic structure passivated both cationic and anionic defects at the grain boundaries, which influenced both the carrier lifetime and the power efficiency of the formed solar cells. Alongside zwitterion molecules, polymers can be also used for the defect passivation. Kojić et al. [40] investigated the influence of polyvinylpyrrolidone as an additive and reported an increase in radiative recombination with smaller Urbach energy values as a consequence of carbonyl interactions with the Pb^2+^ dangling bonds. Inorganic cations can also improve the amount of the trap states, and Ng et al. [41] confirmed this with the inclusion of germanium cations in a quasi 2D/3D perovskite. The thermally stimulated currents with the addition of Ge^2+^ impacted the trap states close to the valence or conduction bands, so perovskite had fewer available states for the electrons to occupy.

A third simulated parameter, the influence of the absorber layer thickness on the cell performance, is studied experimentally by several authors. Rai et al. [42] developed an array of perovskite solar cell devices by varying the active layer thickness with the precursor concentration. In their results, the solar cell efficiency increased with the increase of the perovskite thickness primarily as a result of higher *J*_sc_ values. This is attributed to the reduction of non-radiative decay channels with the increased thickness, and they conclude that thinner absorber layers lead to insufficient photo-generation and increased SRH recombination. Xiao et al. [43] also varied the perovskite thickness with the precursor concentration, reporting maximum *J*_sc_ values for the perovskite thickness of 630 nm. Moreover, they explained that increasing the thickness results in higher current densities as a result of the higher absorption, supported by the external quantum efficiency measurements in the 550–800 nm region. However, increasing the thickness increases the amount of the grain boundaries inside the film, creating charge recombination traps. The authors showed that, by employing a solvent annealing step after the perovskite crystal annealing, it was possible to control the grain size of the film, synthesizing micron thick films with high device efficiency by reducing the amount of grain boundaries.

Furthermore, we investigated the influence of two types of resistance that limit solar cell performance: the net effect of ohmic loss, *R_s_*, and the leakage current loss, *R_shunt_*. Low *R_s_* and high *R_shunt_* are preferred in order to have highly efficient solar cells. The most common contributors to series resistance, *R_s_*, are metal-semiconductor contacts of the solar cells. As the perovskite/hole transport layer/metal contacts are most prone to synthesis variability, influencing their chemical nature and interface can vastly reduce series resistance. The mostly used hole transport layer is the organic material spiro-MeOTAD. Ways to improve the performance of its conductive nature via additives such as tris(2-(1Hpyrazol-1-yl)-4-tert-butylpyridine)cobalt(III) tri[bis-(trifluoromethane)sulfonimide], bis-(trifluoromethane)sulfonimide lithium salt and/or 4-tertbutylpyridine are well established [44]. Rong et al. [45] report the addition of ditetrabutylammonium dichromate in the spiro-MeOTAD hole transport layer by using the four-probe measurements, and found an optimal amount of dichromate addition, which can influence pinhole formation, demonstrates better hole extraction properties and a smaller series resistance compared to the control devices. Guarnera et al. [46] investigated the role of an inorganic Al_2_O_3_ buffer layer between the active layer and the spiro- MeOTAD hole transport layer. In their investigation, they have found that the *V*_oc_ and the *J*_sc_ are similar between the control cells and the buffer layer cells. The most pronounced impact is shown in the *FF* values, with an increase of 25% for cells with the buffer layer. The Al_2_O_3_ buffer layer not only inhibited the formation of a direct contact between the perovskite and the metal contact but showed no degradation during the first 350 h under the full solar illumination. In their investigation of carbon contacts for perovskite solar cells, Babu and co-workers [47] have reported that the use of a thin chromium interlayer between the hole transport layer and the metal (carbon) contact with the thickness of 5 nm provided high shunt resistance and no significant series resistance due to its high ductility. Mundhass et al. [48] have studied the series resistance of the perovskite solar cells using *J*_sc_-*V*_oc_ measurements; in their report, they found that for multi-cation perovskites, the direction of the current sweep (e.g., positive–negative) can impact the value of the series resistance, whereas the series resistance for mono-cation perovskites are independent on the direction of the current sweep. This gives information on how the composition of the perovskite layer can influence the values of the series resistance. In the same paper, they investigated the impact of PTAA hole transport layer thickness on the series resistance and found that the increase of the PTAA thickness reduces the values of *FF*, requiring optimal synthetic approach for a film thin enough to have an optimal *FF* but thick enough not to have pinholes, which can contribute towards shunt resistance. Conversely, there are approaches to film surface interactions by forming interlayers; Veeramutuhu and co-workers [49] used polyvinylpyrrolidone as an interlayer between the perovskite and the hole transport layer, which led to the increase in carrier lifetime with significant reduction of the surface roughness of the perovskite. Hemasiri et al. [50] reported similar results with the utilization of a MoS_2_ interlayer between the perovskite and the hole transport layer; the MoS_2_ interlayer was shown to facilitate the extraction of the photogenerated carriers, mitigating interfacial charge recombination.

A major contribution to low *R_shunt_* are the formation of pinholes and film porosity, and thus several experimental approaches to increase its value are reported in the literature. Brecker and Wark [51] studied the impact of the solvent on the sequential deposition of the perovskite layer. By taking into account solvent permittivity, they have introduced pentan-1-ol into the MAI/isopropanol mixture, which eliminated the pinhole formations in the film and increased the power conversion efficiency by 60%. The usage of a compact TiO_2_ blocking layer below the mesoporous TiO_2_ film has found wide usage in the preparation of the perovskite solar cells, as it provides a way of eliminating shunt resistance issues in combination with the mesoporous TiO_2_. Singh et al. [52] investigated how the thickness of the compact layer impacts the performance of the solar cells; they report an optimal thickness of ~57 nm in order to reduce shunt losses as well as trap-assisted recombination and bimolecular recombination, which become more pronounced with increased compact layer thickness. Hörantner et al. [53] have shown that long alkyl chained silane molecules can be used to block shunt pathways without obstructing the charge transfer of the solar cell devices, leading to the significant increase in the *V*_oc_ values, while other inorganic materials can also be used as passivation materials. Saranin et al. [54] report the use of copper (I) iodide, which has been usually applied as a hole transport layer, as an interlayer between the perovskite and the electron transport layer in their experiments; the inclusion of CuI increased the efficiency of the solar cell as well as their shelf-life stability.

In this work, our intention was to simulate an average perovskite solar cell and use simulation to point out which parameters could be influenced to increase the power conversion efficiency of the device. As a starting point we have synthesized the mixed cation lead triiodide perovskite containing formamidinium, Rb^+^ and Cs^+^ ions (Rb_0.05_Cs_0.1_FA_0.85_PbI_3_) and applied it as an absorber layer in PSC devices with the architecture ITO/TiO_2_/perovskite/spiro-MeOTAD/Au. The obtained *PCE* was around 7%. In order to find routes to increase the *PCE* of the device, we have attempted to simulate the perovskite solar cell by using solar cell capacitance simulator (SCAPS) under standard AM1.5G illumination as a way to understand which parameters can affect its performance and what is the highest *PCE* is that we can theoretically achieve. We used a simulation model to optimize the following parameters influencing the PSC performance: acceptor doping concentrations, defect density, perovskite layer thickness, and resistances: series *R_s_* (net effect of ohmic loss) and parallel *R_shunt_* (the leakage current loss). Moreover, the influence of the interface between layers on PSC performance was discussed in detail.

The main insight of the simulation, showing new input for further optimization of the solar cell efficiency, was finding that the strongest impact on the device performance comes through resistivity of the layers. The negative impact of the resistivity on PSC performances could be improved by influencing layers’ structure quality. Therefore, lowering the density of the defects in layers and interlayers would lower the recombination rate in solar cells.

## 2. Materials and Methods

### 2.1. Materials Used for Preparation of Perovskite Solar Cells

For the synthesis of the Rb_0.05_Cs_0.1_FA_0.85_PbI_3_ perovskite, the following materials were used: lead (II) iodide (Sigma-Aldrich, 99%, St. Louis, MO, USA), formamidinium iodide (FAI) (Sigma-Aldrich, ≥99%, St. Louis, MO, USA), cesium iodide (Sigma-Aldrich, ≥99%, St. Louis, MO, USA), rubidium iodide (Sigma-Aldrich, ≥99%, St. Louis, MO, USA), N,N-dimethylformamide (DMF) (Merck, p.a., Darmstadt, Germany), dimethyl sulfoxide (DMSO) (Merck, p.a., Darmstadt, Germany), chlorobenzene (Merck, p.a., Darmstadt, Germany).

15 × 15 mm indium tin oxide (ITO) coated glass plates (Lumtech, ρ ~15 Ω/sq, CA, USA) were used as substrate and transparent front electrode. For the synthesis of the TiO_2_ ETL titanium diisopropoxide (Sigma-Aldrich, 99%, St. Louis, MO, USA), acetylacetonate (Sigma-Aldrich, 99%, St. Louis, MO, USA) and ethanol (Merck, p.a., Darmstadt, Germany) were used. For the preparation of the spiro-OMeTAD HTL, spiro-OMeTAD (Merck, ≥99%, Darmstadt, Germany), 4-*tert*-butylpyridine (tBP) (Sigma-Aldrich, 99%, St. Louis, MO, USA), FK209 (Sigma-Aldrich, 99%, St. Louis, MO, USA), bis-(trifluoromethane)sulfonimide lithium salt (LiTFSI) (Sigma-Aldrich, 99%, St. Louis, MO, USA) and acetonitrile (Merck, p.a., Darmstadt, Germany) were used.

### 2.2. Preparation and Characterization of Perovskite Solar Cells

In order to prepare the TiO_2_ ETL, 400 μL of acetylacetonate and 600 μL of titanium diisopropoxide were dissolved in 9 mL of ethanol. 50 μL of the prepared solution was spin coated on ITO glass substrates (4000 rpm, 2000 rpm/s) for 30 s and annealed at 450 °C for 2 h.

To prepare the perovskite thin films 461 mg of PbI_2_, 172 mg of FAI, 26 mg of CsI and 106 mg of RbI were dissolved in 1 mL of DMF/DMSO mixture (*V*(DMF):*V*(DMSO) = 4:1). Perovskite films were prepared inside a nitrogen-filled glovebox by spin coating 50 µL of the perovskite precursor on TiO_2_ substrates. The spin coating parameters were: 1000 rpm, 200 rpm/s for 10 s and 6000 rpm, 2000 rpm/s for 20 s. During the last 10 s, 50 µL of chlorobenzene was dripped onto the rotating substrate to improve the nucleation and crystal growth of the film. As prepared substrates were annealed on a hotplate at 150 °C for 10 min.

The spiro-OMeTAD was prepared by dissolving 50 mg of spiro-OMeTAD in 498 μL of chlorobenzene and adding 18 μL of tBP, 10 μL of LiTFSI stock solution and 4 μL of FK209 stock solution. The stock solutions of LiTFSI and FK209 were 1.8 mmol/mL and 0.25 mmol/mL in acetonitrile, respectively. Then 50 µL of the spiro-OMeTAD solution was spin coated on the perovskite/TiO_2_ substrate (4000 rpm, 1000 rpm/s) for 10 s. Before the gold depositions, the substrates were left resting overnight in dark and dry air. The final step included the deposition of 100 nm of gold contacts on the substrates.

Scanning electron microscopy image of prepared PSC was recorded using field emission scanning electron microscope (FE-SEM) model JSM-7000F manufactured by JEOL Ltd., Tokyo, Japan, using an accelerating voltage of 10 kV, equipped with an energy-dispersive X-ray spectrometer (EDS), EDS/INCA 350 (energy-dispersive X-ray analyser) manufactured by Oxford Instruments Ltd., Abingdon, UK.

The current density–voltage (*J–V*) curves of the solar cells were performed using a Keithley 2400 source meter and a LabView-based software inside a glove box (nitrogen atmosphere). For the *J–V* curves, the scan rates were adjusted to 100 mVs^−1^. The illumination area was defined using a shadow mask (0.07 cm^2^) and the light was provided by a Dedolight DLH500 lamp calibrated to an intensity of 100 mWcm^−2^ using a reference silicon solar cell (Fraunhofer ISE). The external quantum efficiency (EQE) spectrum was measured using a MuLTImode 4 monochromator (Amko) equipped with a 75 W xenon lamp and a Keithley 2400 source meter. The monochromatic light was chopped at a frequency of 30 Hz and the measurement setup was spectrally calibrated with a silicon photodiode (Newport Corporation, Irvine, CA, USA, 818-UV/DB). UV–Vis measurements of the perovskite absorber layer were performed using the UV/VIS Spectrometer—Lambda 35 by Perkin Elmer.

### 2.3. Numerical Simulation

Simulation of the solar cell was obtained by using SCAPS-1D software (solar cell capacitance simulator one dimension) which numerically solves one dimensional Poisson and continuity equations that govern the semiconductor material under the steady-state conditions [32]. Poisson equation presents the relationship between the electric field of a p-n junction (*E*) and the space charge density (*ρ*) and is given by:

(1) $$\frac{\partial^{2}\psi }{\partial^{2}x}=-\frac{\partial E}{\partial x}=-\frac{\rho }{\varepsilon_{s}}=-\frac{q}{\varepsilon_{s}}\left[p-n+N_{D}^{+}\left(x\right)-N_{A}^{-}\left(x\right)\pm N_{t}\left(x\right)\right]\;$$

where *ψ* is the electrostatic potential, *q* is the elementary charge, *ε*_s_ is the static relative permittivity of the medium, *n*, *p* are the electron and hole densities, $N_{D}^{+},\;N_{A}^{-}$ are the densities of ionized donors and acceptors and *N_t_* is possible defect (acceptor or donor) density [55]. The electron and hole continuity equations in steady-state are given by:

(2) $$\frac{\partial j_{n}}{\partial x}+G-R_{n}\left(n,p\right)=0$$

(3) $$-\frac{\partial j_{p}}{\partial x}+G-R_{p}\left(n,p\right)=0$$

where *j_n_*, *j_p_* are the electron and hole current densities, *G* is the electron-hole pair generation rate, and *R_n_*, *R_p_* are the net recombination rates of electrons and holes. The electron and hole current densities are given by:

(4) $$j_{n}=qn\mu_{n}E+qD_{n}\frac{\partial n}{\partial x}$$

(5) $$j_{p}=qp\mu_{p}E-qD_{p}\frac{\partial p}{\partial x}$$

where *q* is the elementary charge, *µ_n_*, *µ_p_* are the electron and hole mobility and *D_n_*, *D_p_* are the diffusion coefficients of the electrons and holes. SCAPS-1D software can simulate solar cell structures up to seven layers and extract their basic characteristics, such as the band diagram, the generation and recombination rates, the external quantum efficiency, the cell current densities, the *J–V* characteristic with short-circuit current, open-circuit voltage, fill factor and power conversion efficiency. For SCAPS simulation, the input parameters are listed in Table 1.

## 3. Results and Discussion

**Figure 1:**
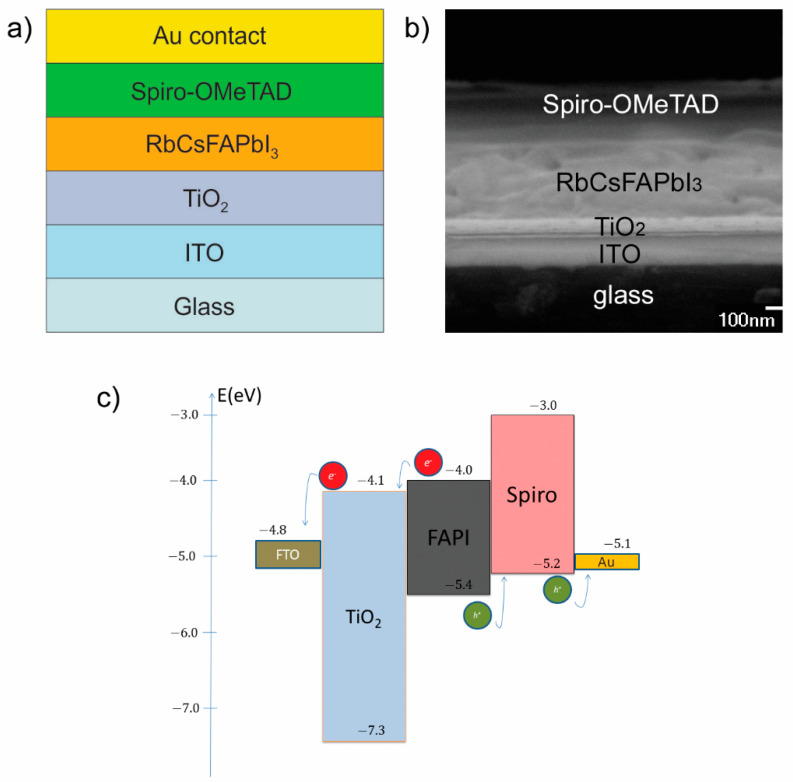
(**a**) The Rb_0.05_Cs_0.1_FA_0.85_PbI_3_ perovskite solar cell layer structure, (**b**) SEM image of layers in our PSC (without gold contact) and (**c**) PSC band diagram.

In the first step of our study, we have simulated the current density–voltage (*J–V*) characteristic and cell performance parameters to obtain a good fit with the *J–V* characteristic measured for our experimentally prepared PSC (Figure 1).

The layer structure of the prepared perovskite solar cell (Rb_0.05_Cs_0.1_FA_0.85_PbI_3_) is illustrated in Figure 1a, its cross-sectional SEM image is shown in Figure 1b, while the energy band diagram is shown in Figure 1c. The energy band diagram (Figure 1c) shows good matching of ETL and HTL with the absorption layer and allows a smooth flow of charge through the cell.

With the aim of increasing the *PCE* of our device, we have studied the influence of the specific parameters on performance of the PSC in order to elucidate which experimental procedure could improve the PSC parameters.

Experimental *J–V* curve was obtained in a two-wire configuration with a Keithley 2400, under 100 mW/cm^2^ illumination. Following, the simulation has been run with the input parameters of the structure summarized in Table 1. They are based on previously published literature [56,57,58,59,60,61] and our experimental results.

Using above-mentioned initial parameters concerning the *J–V* characteristic, simulation results gave the following solar cell performance parameters, which are listed and compared to experimentally obtained data in Table 2.

**Figure 2:**
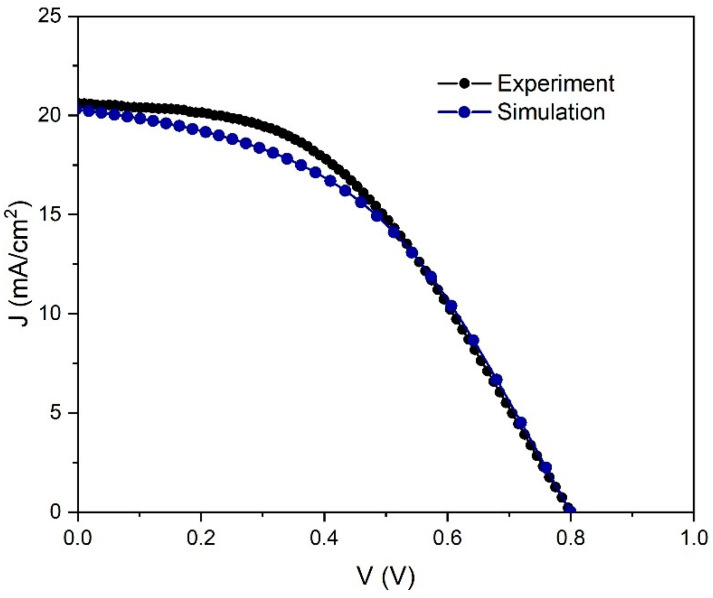
Current density–voltage characteristics under illumination of the experimental and simulated PSC.

Figure 2 shows a comparison between the simulation results and the experiment. A good match between measurements and simulation was observed for the current density/voltage characteristics validating our simulation.

**Figure 3:**
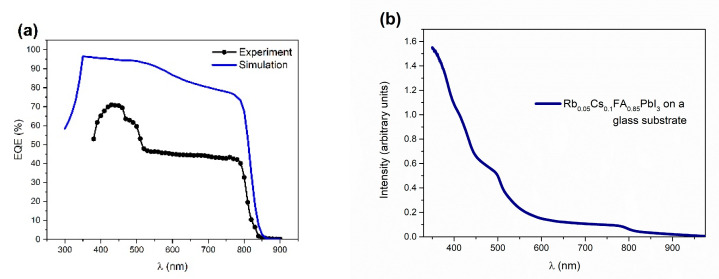
(**a**) EQE (%) spectrum as a result of the simulation with input parameters from Table 1. and (**b**) Absorbance spectra for the Rb_0.05_Cs_0.1_FA_0.85_PbI_3_ perovskite on a glass substrate.

The external quantum efficiency (EQE) spectrum (experiment and simulation), along with the absorbance spectra of the perovskite film, are illustrated in Figure 3. For the simulation of the EQE spectra, the absorption coefficient was calculated from the absorbance spectra. Both EQE curves show the optical absorption edge of the PSC that starts at 800 nm, with the disparity in the lower wavelength region (Figure 3a). Higher experimental values of quantum efficiency on lower wavelength regions are in agreement with the rise in absorbance in the same region (Figure 3b). However, as the mechanism of this experimentally observed behavior is unclear, except for a gradual increase in the EQE values, the simulated spectra could not completely fit the experimental data.

### 3.1. Doping Concentration Impact of Perovskite Active Layers on PSC Performance

**Figure 4:**
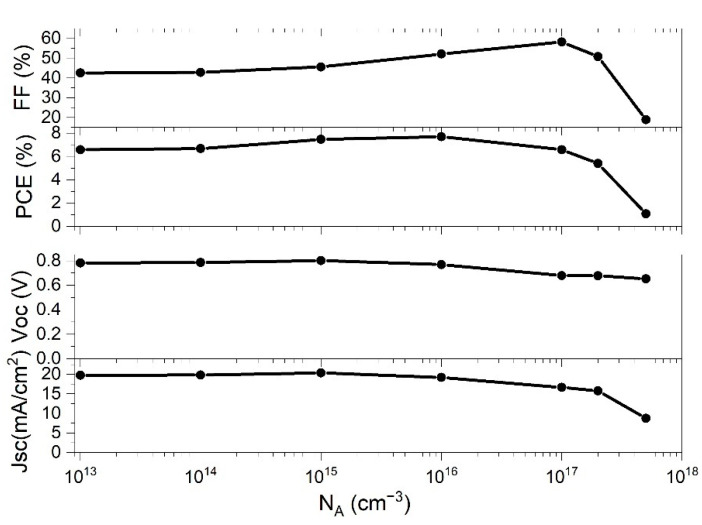
Effect of changing doping concentration (*N*_A_) of absorber layer on PSC parameters.

In order to study how the concentration of *N*_A_ of the perovskite thin film impacts the performances of the solar cell, we have investigated *N*_A_ values in the range from 10^13^ cm^−^^3^ to 5·10^17^ cm^−^^3^ in the simulation. The rest of the parameters were fixed to values indicated in Table 1. Figure 4 shows the behavior of the PSC parameters with the change of *N*_A_.

**Figure 5:**
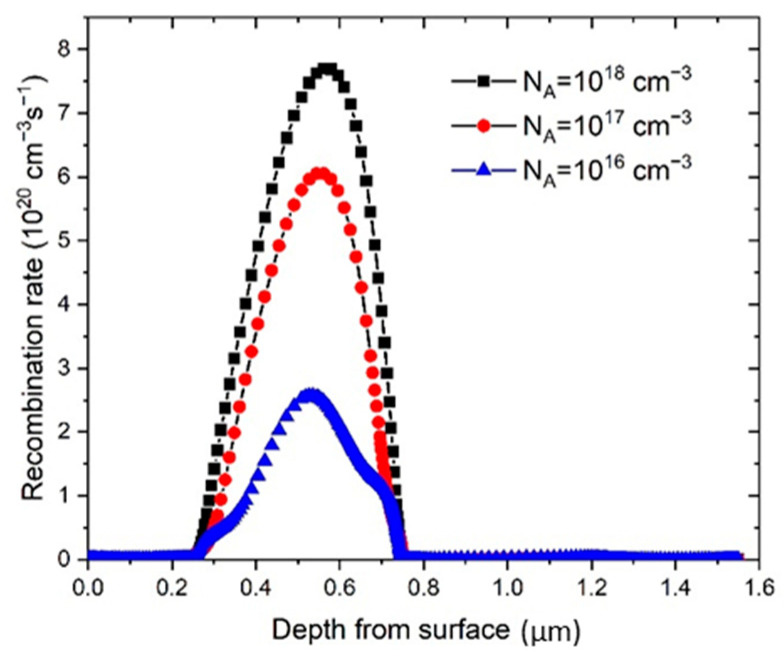
Change of recombination rate with *N*_A_ of the perovskite. Zero depth value corresponds to the substrate (ITO surface).

The *FF* slowly raised with the enhancement of *N*_A_, and then rapidly decreased after *N*_A_ went beyond 10^17^ cm^−^^3^. *PCE* exhibited similar behavior, having a maximum value at *N*_A_ = 10^16^ cm^−^^3^, and then rapidly decreased. The other two parameters, *J*_sc_ and *V*_oc_ are also influenced by the change of *N*_A_, both of which have their local maximum at ~10^15^ cm^−^^3^. At higher *N*_A_ concentrations, *V*_oc_ decreased slowly, while *J*_sc_ decreased very rapidly after *N*_A_ exceeded 10^17^ cm^−3^. The difference of the cell performance in respect to the *N*_A_ can be explained by the rise of the built-in electric field with the increased concentration of the *N*_A_ [16,24]. Charge carriers separation is more pronounced with an increased electric field and consequently improves the performance of the cell. However, even larger values of *N*_A_ will lead to higher Auger recombination rates and subsequently PSC parameters will decrease [62]. As it can be seen in Figure 5, the recombination rate, *R*, is increased notably, starting with *N*_A_ values of 10^16^ cm^−^^3^, which is in good correlation with PSC parameters presented in Figure 4.

Therefore, only a range of *N*_A_ values can contribute to the improvement of *J*_sc_ and *V*_oc_ and the increase the *PCE* value. The deposited perovskite films optimally have low charge carrier concentration in order to maximize the carrier mobility inside the perovskite thin film. At the *N*_A_ values of 10^16^ cm^−3^, the maximum *PCE* of 7.71% was obtained, while the other parameters of PSC for that N_A_ concentration were *V*_oc_ = 0.77 V, *J*_sc_ = 19.23 mA and *FF* = 52.07% (see Figure 4). The result of the simulation shows that our starting value for N_A_, from the parameter set which optimally represents the experimental data (Table 1), is very close to the optimal value obtained by theoretical modelling (*N*_A_ = 10^16^ cm^−3^).

Generally, doping concentration of the active layer in PSC could be optimized experimentally by changing synthesis precursor concentrations, solvent choice [33], changing the length of thermal annealing [34], or by adding the cations or the anions in the perovskite active layer [35,36], as was stated in detail in the introduction.

### 3.2. Defect Density Impact on PSC Performance

Regarding the improvement of the PSC performance, the defect density is an additional parameter that should be taken into account. The morphology and film quality of the perovskite have been shown to have an important influence on the performance of the perovskite solar cell [63]. Poor quality and film coverage on mesoporous TiO_2_ has shown to increase the charge recombination inside the active layer [64], and the recombination is explained by the increase in the defect density (*N_t_*), which can impact the *V*_oc_ of the solar cell.

The Shockley–Read–Hall recombination model (SRH) was used to study the influence of the perovskite active layer defect density on the cell performance. The neutral defects were set at the center of the band gap following the Gaussian distribution with the characteristic energy value of 0.1 eV, and the characteristic energy of 0.1 eV were set to be at the center of the band gap. In the SRH recombination model, the recombination rate, *R*, is defined as [65,66]:

(6) $$R=\frac{np-n_{i}^{2}}{\tau_{p}\left(n+N_{C}e^{\left(E_{c}-E_{t}\right)/kT}\right)+\tau_{n}\left(p+N_{V}e^{\left(E_{t}-E_{v}\right)/kT}\right)}$$

where *n* is the concentrations of the mobile electrons and *p* is the concentrations of the holes. The concentrations of the charge carriers can be found by solving Poison and continuity equations; at positive voltage values, where *qV >* 3 *kT*, the term *n_i_*^2^, which explains the thermal generation, can be neglected. *E_t_* is energy level, while *N_t_* is the concentration of the trap defects; *τ_n_* is the lifetime for the electrons, while *τ_p_* is the lifetime of the holes. They are given by equations:

(7) $$\tau_{n}=\frac{1}{\sigma_{n}v_{th}N_{t}},\tau_{p}=\frac{1}{\sigma_{p}v_{th}N_{t}},$$

where *σ_n_* is the capture cross-sections of the electrons, *σ_p_* is the captured cross-sections of the holes, while *v_th_* is the thermal velocity.

The carrier diffusion length, *l*, is is defined by the equation:

(8) $$l=\sqrt{D\tau },$$

where *D* is the diffusion coefficient with the equation:

(9) D=μkT/q

where *μ* is the charge carrier mobility. As Equation (7) indicates, when defect density decreases, the charge carrier lifetimes increase, leading to longer diffusion lengths (Equation (8)) and lower recombinations. These were the main factors influencing the improvement of the cell performance.

**Figure 6:**
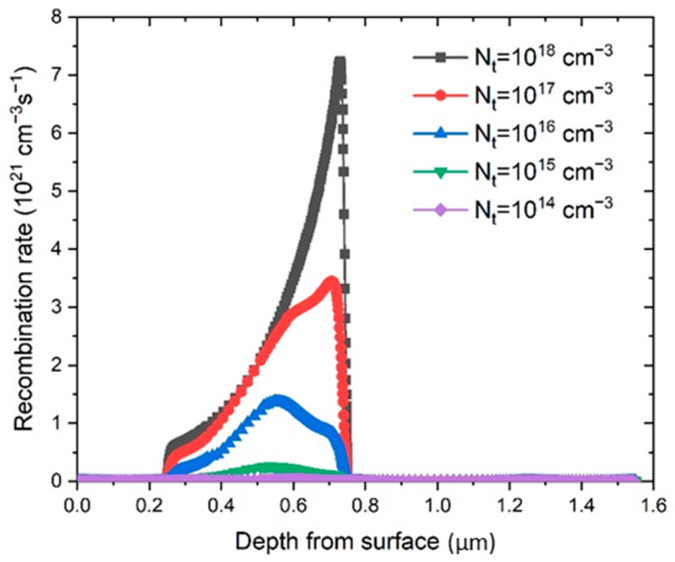
Recombination rate variance as a function of depth starting from ITO layer surface for different *N_t_* values.

Defect density, *N_t_*, was investigated as a parameter in the PSC performance, and the defect density values were changed from 10^14^ cm^−3^ to 10^18^ cm^−3^. The change in the recombination rate (*R*) (Equation (6)) is shown in Figure 6. It is clear that the reduction of *N_t_* lowers the recombination rate, and at the same time increases the diffusion length, *l*.

**Figure 7:**
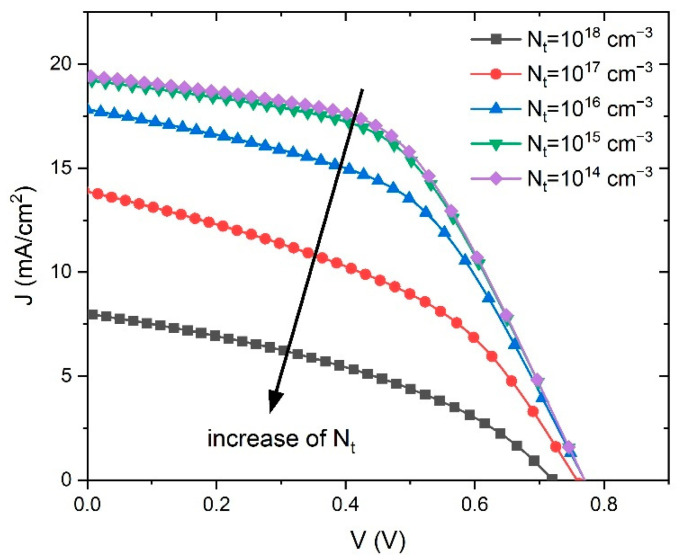
Current density–voltage curves curves of simulated PSCs as a function of defect density *N_t_*.

Therefore, the defect density reduction in the perovskite significantly improved the performance of the PSC, which is consistent with the simulation results showed in Figure 7. The obtained simulated *J–V* characteristics show an improvement of the PSC parameters with the reduction of *N_t_*.

The lowest defect density of 1·10^14^ cm^−3^ gives the best performance, but obtaining such a low *N_t_* in experiments is very difficult due to the polycrystalline nature of the perovskite films, and thus we set the optimized value of defect density at 1·10^15^ cm^−3^. As can be seen from Table 1, that value was also the initial value of *N_t_*, which gave the best fit to the experimental voltage-V curve.

The defects can be interpreted as the faults inside experimentally obtained solar cells due to the grain boundaries or as dangling bonds, uncoordinated atoms, and surface dislocation on the film surface. As was presented in introduction, the defect density can be decreased experimentally by post annealing with the aim to obtain oriented crystal growth [37,38], with additive engineering [39,40], or by the inclusion of cations which impacted the trap states [41].

### 3.3. Impact of the of the Perovskite Active Layer Thickness on the PCS Performance

**Figure 8:**
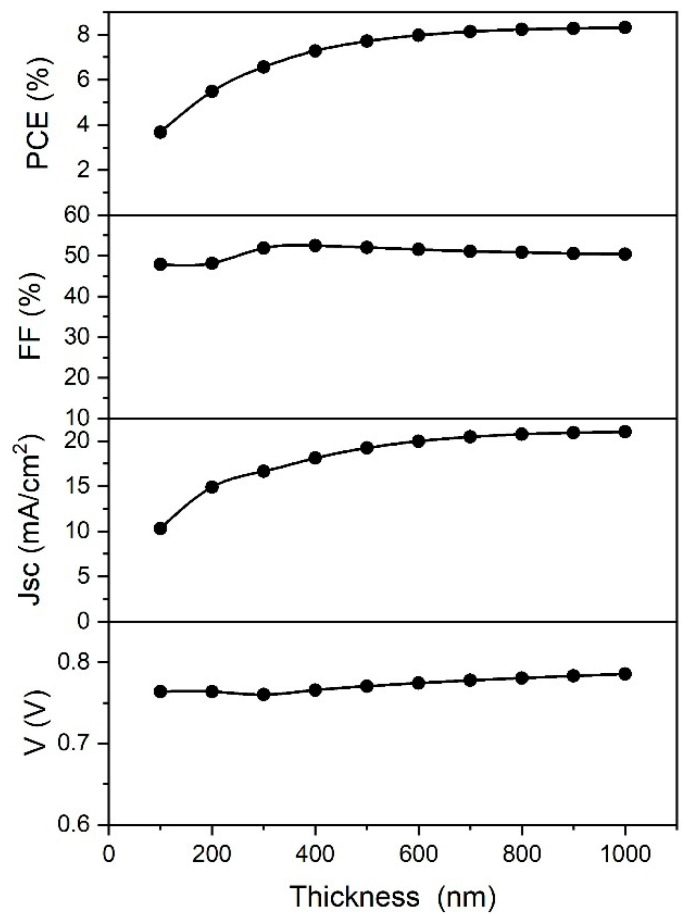
Variation of PSC parameters with the perovskite layer thickness.

Absorbing layer thickness is another parameter that plays a very important role in the performance of thin film solar cells. The influence of the absorber layer thickness on the cell performance with is shown in Figure 8.

When the thickness is too low, the absorption of the light is also very low, giving lower *PCE* values. As the thickness of the absorber is increased, the absorption of the light is increased as well, improving the *PCE*. With the thickness values larger than 800 nm, the *PCE* of the cell remains invariable. However, an increase in thickness leads to larger recombination rates in the bulk (depending on the diffusion length), saturating the *PCE* and *J*_sc_ values. This is the reason why the *PCE* shows a similar behavior to the *J*_sc_. The other two parameters, *FF* and *V*_oc_, are not affected significantly by change of the absorber thickness.

Experimentally, the active layer thickness could be varied with the variation of precursor concentration [42,43], as is reported in the introduction.

### 3.4. Influence of Resistivity R_s_ and R_shunt_ on PSC Performance

**Figure 9:**
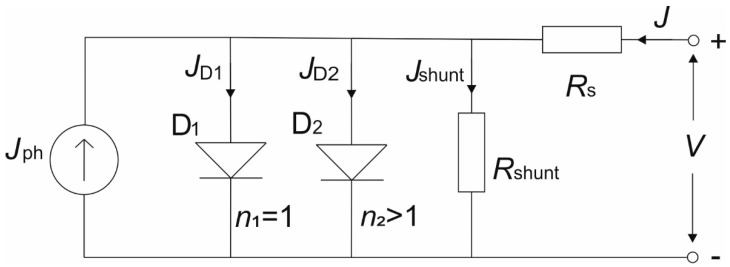
The equivalent circuit model of solar cell: *J*_ph_ is photogenerated current, D_1_ and D_2_ are ideal and non-ideal diode with ideality factors *n*_1_ = 1 and *n*_2_ >1 and currents *J*_D1_ and *J*_D2_, respectively. *R_s_* and *R_shunt_* represent series and parallel resistances with currents *J* and *J*_shunt_, respectively.

Under illumination, an ideal solar cell can be represented by a parallel circuit which consists of an ideal diode (*D*_1_) and a constant current source of photogenerated current (*J*_ph_). The improved equivalent circuit model, so called 2-diode model, is shown in Figure 9 [67,68]. Equivalent circuit for solar cell under dark condition differs only in vanishing photogenerated current source J_ph_.

The ideal diode *D*_1_ represents the radiative recombination in the solar cell (perovskite layer), while the non-radiative recombination processes are represented by the diode *D*_2_. In the equivalent circuit, two resistances are present: series *R_s_* and parallel *R_shunt_*, which have a high impact on solar cell performance, since they govern the shape and slopes of the *J–V* characteristics. They can be calculated from the slope of the *J–V* curve at the *V*_oc_ point (*R_s_*) and at *J*_sc_ point (*R_shunt_*). *R_s_* represents the net effect of the ohmic loss and has its origin in the electrical resistance of contacts (ITO and gold in our PSC) and in the electrical dissipation, which is occurring in the layers of PSC (ETL, perovskite and HTL). In contrast, *R_shunt_* represents the leakage current loss induced by defects in the solar cell, such as traps and pinholes. *R_s_* mainly affects the *FF* and *J*_sc_, while a low *R_shunt_* value results in a photovoltage loss and can affect the collected photocurrent. It is commonly known that a low *R_s_* and high *R_shunt_* are preferred in order to have highly efficient solar cells. The *J–V* characteristic of the equivalent circuit can be described by the following equation [36]

(10) $$J=J_{1}\left(e^{\frac{q\left(V-AJR_{s}\right)}{n_{1}k_{B}T}}-1\right)+J_{2}\left(e^{\frac{q\left(V-AJR_{s}\right)}{n_{2}k_{B}T}}-1\right)+\frac{V-AJR_{s}}{R_{shunt}}-J_{ph}$$

where *J*_1_ and *J*_2_ are the saturation current densities of the two diodes, respectively, *A* is the area of the solar cell, *n*_1_ and *n*_2_ are the ideality factors of the two diodes.

**Figure 10:**
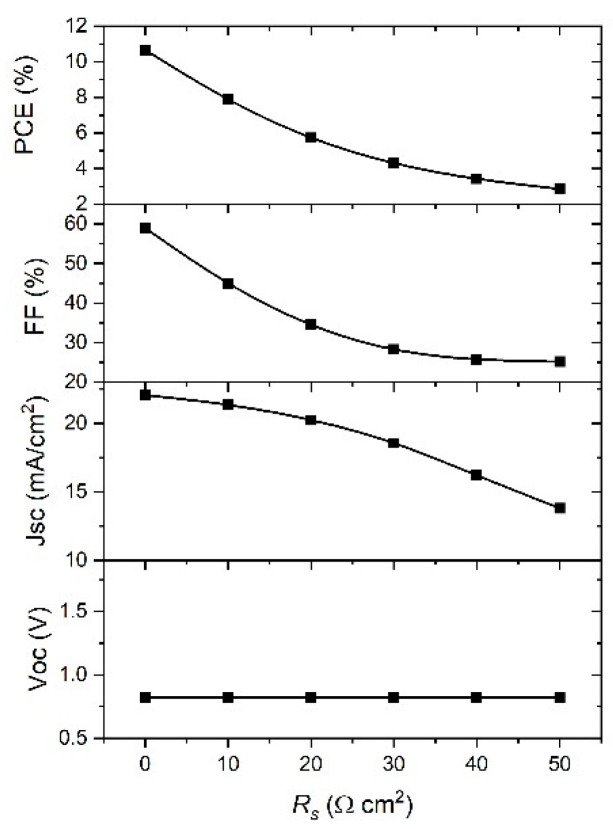
Effect of *R_s_* on the PSC parameters.

**Figure 11:**
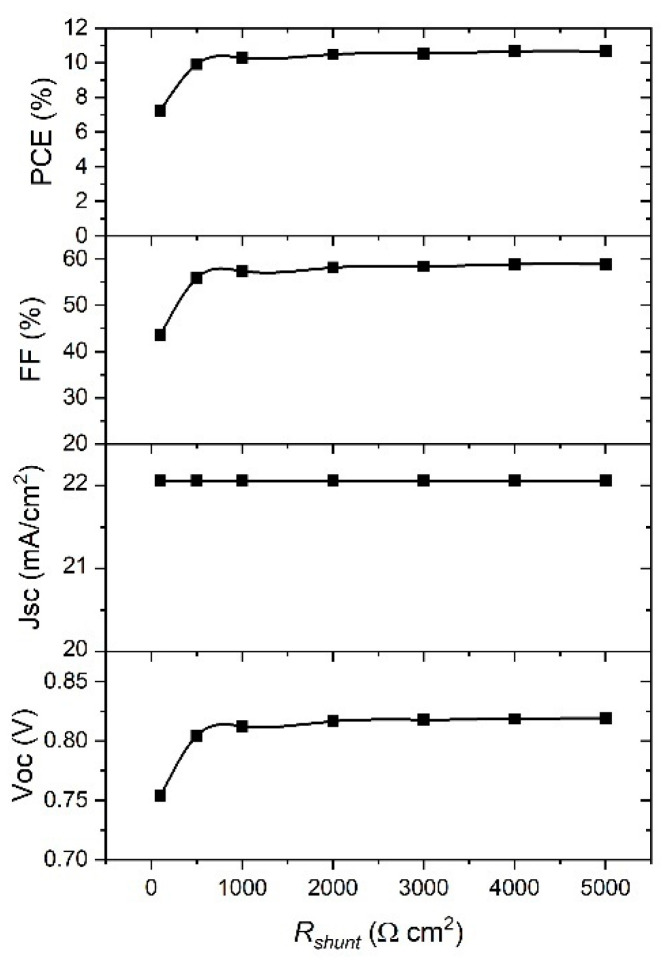
Effect of *R_shunt_* on the PSC parameters. *R_shunt_* is assumed as infinite in this case.

In order to understand the effect of *R_s_* and *R_shunt_* on the performance of PSCs, the SCAPS model was used with the range of their values 0–50 Ω cm^2^ and 100–5000 Ω cm^2^, respectively. The obtained simulation results are given in Figure 10 and Figure 11, and it is clear from the graph in Figure 10 that the *V*_oc_ is not affected by the change of *R_s_*, while other parameters decreased with the increase in *R_s_* value; indeed, the *J*_sc_ decreases slowly from 22 mA cm^−2^ to less than 15 mA cm^−2^. The *FF* value was reduced by half, from 60% to less than 30%, while the *PCE* decreased from 10% to approximately 3%. It can be observed from the results that PSC parameters are strongly affected by the change of *R_s_*, except the *V*_oc_, meaning the best device performance will be for the lowest *R_s_*.

Considering the dependence of the PSC parameters on *R_shunt_*, Figure 11 clearly shows that *J*_sc_ is the parameter that is not affected by *R_shunt_* change. The rest of parameters show a significant increase when *R_shunt_* increased from 100 to 1000 Ω cm^−2^, and they are saturated for higher values of *R_shunt_*. Therefore, the higher values of *R_shunt_*, the better performance of the device. From the best fit of the experimental *J–V* curve (Figure 2) we obtained the values for *R_s_* = 9 Ω cm^−2^ and *R_shunt_* = 1000 Ω cm^−2^. When we used the ideal values of *R_s_* and *R_shunt_*, and ran a simulation of our device, we achieved a significant improvement in *PCE* from 7.25% to 10.8%.

**Figure 12:**
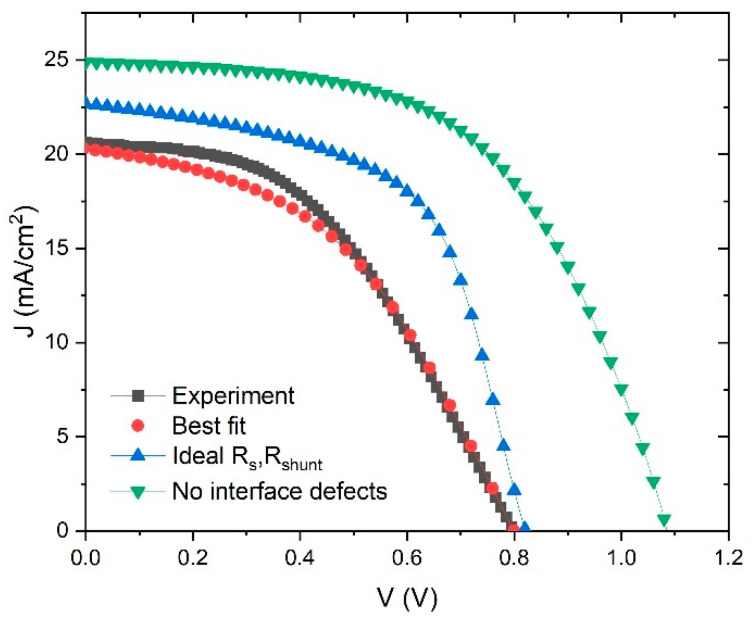
*J–V* characteristics: experimental and best fit (*R_s_* = 9 Ω cm^−2^, *R_shunt_* = Ω cm^−2^, ideal *R_s_*, *R_shunt_* (*R_s_* = 0 Ω, *R_shunt_* = infinite) and no intefrace defects (removing influence of interface defects with *R_s_* = 0 Ω, *R_shunt_* = infinite).

There is one more issue in SCAPS model which can significantly affect the performance of the device through *R_s_* and *R_shunt_*, and this is the interface layer between ETL-perovskite and perovskite-HTL. The interface layer is a source of high concentration of defects, which can drastically decrease the device performance. In many studies [56,61], the initial parameter of defect concentration in interface layers is usually set around *N_t_* = 10^16^–10^17^ cm^−3^. We can simulate the reduction in defect concentration and study its influence on performance of the device, and this simulation can be seen in Figure 12.

First, Figure 12 shows the experimental and best fit *J–V* curves, as in Figure 2. In addition, there is a curve that describes an improvement in device parameters by taking the ideal values of *R_s_* and *R_shunt_* (*V*_oc_ = 0.82 V, *J*_sc_ = 22.7 mA/cm^2^, *FF* = 58.2%, *PCE* = 10.8%). We can improve these parameters even further by taking an ideal structure (no interface defects), but only for academic reasons, to see the theoretical limit of the device performance. In that case, the PSC parameters are: *V*_oc_ = 1.09 V, *J*_sc_ = 24.9 mA/cm^2^, *FF* = 56.1% and *PCE* = 15.17%. This means that, theoretically, our device has enough space to double its *PCE* from 7.25% to 15%. One way to ensure better performance is to lower resistivity and decrease interface defects by improving structures of the device layers.

Therefore, it is possible to improve the PSC performances experimentally, mainly by influencing *R_s_* and *R_shunt_*. Experimentally, series resistance, *R_s_*, can be decreased by additives in HTL [44,45], by inserting the interlayers in PSCs [46,47,49,50], or by the composition of the perovskite layer [48].

Since low *R_shunt_* is a consequence of pinholes formation and film porosity, it can be increased experimentally by solvents’ additives which eliminate formation of pinholes [51], by the usage of a compact TiO_2_ blocking layer below the mesoporous TiO_2_ film [52], or by passivation materials that block shunt pathways [53,54].

## 4. Conclusions

SCAPS-1D software was used to successfully simulate the *J–V* characteristics of the experimentally measured perovskite solar cell with an ITO/TiO_2_/Perovskite/Spiro-MeOTAD/Au structure. The values obtained by modelling (*J*_sc_ = 20.29 mA/cm^2^, *V*_oc_ = 0.80 V, *FF* = 44.68% and *PCE* = 7.25%) were similar to measured values (*J*_sc_ = 20.60 mA/cm^2^, *V*_oc_ = 0.8 V, *FF* = 45.51% and *PCE* = 7.35%).

Acceptor doping concentrations of the perovskite layer were optimized by simulations of the value *N_A_* = 10^16^ cm^−3^. As a result of the simulation, when the thickness increased above 800 nm, the PCE remained invariable.

It was shown that the *V*_oc_ is not affected by a change in *R_s_*, while the other parameters decreased with the increase of *R_s_*, so the best device performance will be for the lowest *R_s_*. Regarding the *R_shunt_*, *J*_sc_ is not affected by *R_shunt_* change, while the rest of the parameters show a significant increase when *R_shunt_* rose from 100 to 1000 Ω cm^2^, and they are saturated for higher values of *R_shunt_*. 

The reduction of the defect concentration in the interface between layers was also simulated to see its influence on the performance of the device, by taking the ideal values of *R_s_* and *R_shunt_* (no interface defects), so the theoretical limit of the device performance was obtained: *V*_oc_ = 1.09 V, *J*_sc_ = 24.9 mA/cm^2^, *FF* = 56.1% and *PCE* = 15.17%. Therefore, theoretically, our device could double its *PCE* from 7.25% to 15%.

The results have shown that SCAPS-1D software can be used to adequately simulate non-ideal perovskite solar cells. In this regard, SCAPS can be used as a tool to study potential failure points of obtained perovskite solar cell and as a guide where and how to improve the device performance. Furthermore, the results have shown that, for the PSC experimentally prepared in this work, we can improve *PCE* mainly by influencing *R_s_* and *R_shunt_*. Taking into account the thermal stability of the material, the Rb_0.05_Cs_0.1_FA_0.85_PbI_3_ perovskite is a very promising material for further studies.

## Figures and Tables

**Table 1:** Perovskite solar cell input parameters.

Parameter	ITO	TiO_2_	Rb_0.05_Cs_0.1_FA_0.85_PbI_3_	spiro-OMeTAD
Thickness (nm)	300	100	500	550
Band gap (eV)	3.6	3.26	1.55	2.9
Electron affinity (eV)	4.1	4.2	3.95	2.2
Dielectric permittivity	10	10	6,6	3
CB effective density of states (cm^−3^)	2·10^18^	2.2·10^18^	2·10^19^	2.2·10^18^
VB effective density of states (cm^−3^)	1.8·10^19^	1.8·10^18^	2·10^18^	1.8·10^18^
Thermal velocity of electrons (cm/s)	10^7^	107	10^7^	10^7^
Thermal velocity of holes (cm/s)	10^7^	107	10^7^	10^7^
Electron mobility (cm^2^/Vs)	50	20	0.28	10^−4^
Hole mobility (cm^2^/Vs)	75	10	2	10^−4^
Shallow donor density *N*_D_ (cm^−3^)	10^19^	10^17^	0	0
Shallow acceptor density *N*_A_ (cm^−3^)	0	0	1.3·10^16^	10^18^
Defect density *N_t_* (cm^−3^)	10^15^	10^15^	10^15^	10^15^

**Table 2:** Experimental and simulated parameters of the PSC: short-circuit current density (*J*_sc_), open-circuit voltage (*V*_oc_), fill factor (*FF*) and power conversion efficiency (*PCE*).

Parameter	Experimental	Simulated
*V*_oc_ (V)	0.80	0.80
*J*_sc_ (mA/cm^2^)	20.60	20.29
*FF* (%)	45.51	44.68
*PCE* (%)	7.35	7.25

## Data Availability

Data is contained within the article.
